# Exploring the outcomes of psychotherapy sessions: how do therapists' responsiveness and emotional responses to patients with personality disorders affect the depth of elaboration?

**DOI:** 10.3389/fpsyg.2024.1390754

**Published:** 2024-09-05

**Authors:** Flavia Fiorentino, Ivan Gualco, Antonino Carcione, Vittorio Lingiardi, Annalisa Tanzilli

**Affiliations:** ^1^Department of Dynamic and Clinical Psychology, and Health Studies, Faculty of Medicine and Psychology, Sapienza University of Rome, Rome, Italy; ^2^Center for Individual and Couple Therapy, Genoa, Italy; ^3^Third Center of Cognitive Psychotherapy, Rome, Italy

**Keywords:** psychotherapy process, session outcome, depth of elaboration, responsiveness, therapist emotional responses, TRQ, PEAR, SEQ

## Abstract

**Background:**

The impact of depth of elaboration in individual psychotherapy sessions on overall treatment effectiveness was found in the empirical literature. In the best sessions, relevant content is processed with greater depth; in contrast, in the shallower sessions, the emerging content is more superficial. Evidence suggests that achieving a high level of depth is closely related to specific therapist characteristics and relational dimensions (including clinicians' emotional responses to patients). The present study aimed to (a) compare therapist responsiveness and countertransference patterns in psychotherapy sessions with different levels of depth of elaboration; and (b) examine if the positive countertransference pattern mediated the relationship between therapist responsiveness and depth of elaboration.

**Methods:**

Eighty-four clinicians were asked to select one patient with personality disorders in their care and complete the *Depth Scale of the Session Evaluation Questionnaire*, the *Patient's Experience of Attunement and Responsiveness Scale*, and the *Therapist Response Questionnaire* concerning one of their sessions.

**Results:**

The results showed that sessions with higher levels of depth of elaboration were characterized by greater therapist responsiveness and more positive countertransference. Conversely, poor therapist responsiveness and hostile/angry, disengaged, and helpless/inadequate countertransference responses were found in shallower sessions. Moreover, positive countertransference mediated the relationship between therapist responsiveness and depth of elaboration.

**Conclusion:**

This study sought to shed light on the processes underlying the outcomes of psychotherapeutic sessions, highlighting the strong impact of relational factors. Advancing knowledge of these mechanisms seems crucial to identifying the active ingredients of the therapeutic process and understanding what (does not) promote successful outcomes.

## 1 Introduction

The effectiveness of psychotherapies across diverse clinical populations has been widely established in the empirical literature (Cuijpers et al., 2013, 2014; Cristea et al., 2017). However, evidence indicates that not all patients benefit from psychological treatment, with some reporting unhelpful—and sometimes even harmful—effects (Mohr, 1995; Castonguay et al., 2010a; Scott and Young, 2016). Currently, the role and interaction of complex factors—including therapist variables such as their interpersonal skills (e.g., Lingiardi et al., 2018)—that shape the treatment process and contribute to both psychotherapy successes and failures remain partially unclear (Mulder et al., 2017; Norcross and Lambert, 2018). This suggests the need for a specific focus on how treatment works—that is, on the underlying mechanisms of change—rather than limiting evaluations to their efficacy (Kazdin and Nock, 2003; Cuijpers et al., 2019). Notably, the treatment of individuals with personality disorders diagnoses represents a challenge for clinicians. This clinical population's highly impaired interpersonal patterns (Wilson et al., 2017) demand highly sophisticated therapist interpersonal skills and planning of individualized interventions (Caligor et al., 2015; Kramer, 2021; Kramer et al., 2022a).

Exploring the process of individual therapy sessions—that is, the “session's immediate subjective effect on patients' reactions” in terms of interpersonal climate, sense of progress and satisfaction (Stiles et al., 2002, p. 326)—may help gain a more refined understanding of underlying mechanisms of change related to long-term treatment (in)effectiveness (Orlinsky and Howard, 1968; Gelo and Manzo, 2015; Kramer et al., 2020). Events within each session have an impact on the events occurring in other sessions and in the whole treatment, offering valuable insights into the ongoing therapeutic process and, consequently, on long-term psychotherapy outcomes (Stiles, 1980; Stiles et al., 1988; Lingiardi et al., 2011).

The “curative” effect of exploring deep contents in therapeutic sessions has been emphasized (Greenberg and Pascual-Leone, 2006). Stiles and Snow (1984a,b) have specifically investigated the dimension of depth of elaboration as a critical factor in defining the quality of psychotherapy sessions. In negative sessions, perceived as shallow, weak, worthless, empty, and ordinary, the therapeutic dyad tends to address topics superficially and concretely, with little focus on patients' emotions (Stiles et al., 2002). Conversely, good sessions which are deep, powerful, valuable, full, and special, typically foster a sense of safety (cf., Mallinckrodt et al., 2005), creating an environment conducive to the exploration of interpersonal problems, conflicts and, more broadly, psychological issues related to inner dynamics of the patient (Stiles, 1988; Lingiardi et al., 2011). Notably, several findings (e.g., Mallinckrodt, 1993; Reynolds et al., 1996; Samstag et al., 1998; Rocco et al., 2017), including meta-analytic estimates (Pascual-Leone and Yeryomenko, 2017), have emphasized that attaining higher levels of depth of elaboration is associated with positive treatment outcomes, supporting the need for further investigations into factors that might facilitate or hinder this in-session process.

In this regard, the therapist's interpersonal characteristics were believed to influence the therapeutic relationship and the ongoing psychotherapy (Norcross and Lambert, 2019). However, knowledge about their function as mechanisms of change at the in-session level is scarce. One dimension that has recently received significant attention from the scientific community is therapist responsiveness (Elkin et al., 2014; Kramer and Stiles, 2015; Snyder and Silberschatz, 2017; Wu and Levitt, 2020). Described as a complex and ubiquitous capacity inherent to all human relationships, responsiveness emerges from the moment-to-moment interaction (Stiles et al., 1998). Snyder and Silberschatz (2017) describe an attuned and responsive clinician as someone who deeply understands the patient's emotional state, creating a warm, safe, and genuine environment reminiscent of a mother-child relationship (cf., Stern et al., 1984). These clinicians continually seek to adapt their intervention to meet the specific needs of their patients by engaging in a process of mutual and interactive regulation (Constantino et al., 2013; Owen and Hilsenroth, 2014; Anderson et al., 2020). Being responsive toward patients presenting with personality pathologies can be especially difficult (Kramer, 2021). These patients have severe deficits in interpersonal functioning that strongly impact the therapeutic relationship, making it difficult for clinicians to be adequately attuned to their needs, especially when they show anger, hostility, and impulsive or dangerous behaviors (McMain et al., 2015; Culina et al., 2023, 2024). Responsiveness has been linked to positive treatment outcomes in different clinical populations (Hardy et al., 1998; Elkin et al., 2014), including patients with personality disorders (McMain et al., 2015; Signer et al., 2020; Kramer et al., 2022b). To date, only the study by Harrington et al. (2021) has specifically examined the relationship between responsiveness and the depth of elaboration. The authors emphasized the importance of a responsive attitude, particularly with patients who struggle to engage in profound and meaningful topics during the first phase of the therapy. They showed greater benefit when their therapist responsively focused on these issues, reporting less interpersonal distress.

Another critical factor that can significantly influence the outcome of psychotherapy is countertransference (or, in this context, therapist responses or reactions) (cf., Hayes et al., 2015, 2018; Abargil and Tishby, 2022). Currently, it is largely recognized as a valuable tool that can sensitively inform the diagnostic and therapeutic process (Gabbard, 2001; cf., Tanzilli and Lingiardi, 2022). In a broader or *totalistic* view (Kernberg, 1965), countertransference is defined as the clinician's whole range of feelings, thoughts, and behaviors toward the patient. Especially, negative emotional responses may be challenging to manage (cf., Gross and Elliott, 2017; Tanzilli et al., 2021; Hennissen et al., 2023), potentially obstructing the exploration of meaningful topics within sessions, moving away the therapist's focus from the patient's issues (Abargil and Tishby, 2022; Pellens et al., 2023). Notably, individuals with personality disorders often elicit intense emotional responses in their clinicians, such as feelings of inadequacy or overwhelm, “re-actualizing” their dysfunctional relationship patterns in the therapeutic relationship (Betan et al., 2005; Tanzilli et al., 2016). Research indicated limited and inconsistent results on the relationship between depth of elaboration and countertransference, highlighting a gap in the literature. For example, Rocco et al. (2021) investigated the relationship between the therapist's emotional responses and depth without finding significant associations. In another research, Rosenberger and Hayes (2002) emphasized the importance of adequate countertransference management to promote deeper elaboration in psychotherapy sessions, while Markin et al. (2013) found that positive countertransference behaviors were associated with smoother sessions. In addition, decreased depth over time in therapists was associated with increased positive emotional expression (Markin et al., 2013; Hayes et al., 2018). These results signal the urgent need for further studies to overcome these field gaps.

Finally, several empirical investigations (Ulberg et al., 2014; Tishby and Wiseman, 2022; Hennissen et al., 2023; Pellens et al., 2023) have shown that negative countertransference responses can affect the therapist's capacity to be appropriately emphatic and attuned, and to provide accurate interventions (i.e., to be responsive) in the clinical work. For instance, Hennissen et al. (2023) stressed the importance of dealing with feelings of disengagement toward self-critical patients. If not adequately handled (Hayes et al., 2018), these reactions can challenge the ongoing care process, hindering the construction of a collaborative environment (Gross and Elliott, 2017; Tanzilli and Gualco, 2020). Moreover, although evidence indicating therapist responsiveness seems to foster feelings of trust and collaboration through the treatment process (Elkin et al., 2014; Hatcher, 2021), the possible impact on positive countertransference—indicative of a connection within the therapeutic dyad and a perception of competency in working with the patient—has not yet been investigated (Tanzilli et al., 2016).

Overall, evidence supports the role of depth of elaboration as a marker of psychotherapy session quality and treatment effectiveness (Stiles et al., 2002). However, there is still limited knowledge about the impact of therapist's and relational variables on this dimension of individual session outcomes in the treatment of patients with personality disorder diagnoses. With these premises in mind, the present research aimed to:

(a) examine differences in therapist responsiveness and countertransference patterns in sessions with higher or lower levels of depth of elaboration, regardless of the duration of treatment. Based on the clinical and empirical literature (Stiles et al., 2002; Tishby and Wiseman, 2014; Harrington et al., 2021; Pellens et al., 2023), it was expected that poorer clinician responsiveness and more intense and negative patterns of therapist emotional responses would have a greater impact on the shallower sessions, net of the effect of the length of therapy; conversely, higher levels of responsiveness and more positive countertransference reactions would significantly affect sessions characterized by a greater degree of depth;

(b) through exploratory analysis, to investigate whether the therapist's positive emotional responses would mediate the relationship between clinician responsiveness and depth of session processing. Despite the paucity of studies in this field of investigation (Ulberg et al., 2014; Tanzilli et al., 2016, 2018; Pellens et al., 2023), positive countertransference would be a significant mediator that could partially account for the effect of clinician responsiveness on good session quality.

## 2 Materials and methods

### 2.1 Participant sampling

The sample of therapists was recruited from several Italian associations of psychodynamic and cognitive-behavioral psychotherapy and centers specializing in treating personality disorders in Genoa, Milan, Turin, and Rome. They were contacted via email and asked to identify one patient in their care according to the following inclusion/exclusion criteria: (1) at least 18 years old; (2) a personality disorder diagnosis [according to the DSM-5/5-TR (APA, 2013, 2022) or ICD-10/11 (WHO, 1993, 2022)] (3) without psychotic disorder diagnosis, nor treated with pharmacological therapy for psychotic symptoms; (4) in treatment from a minimum of 2 to a maximum of 12 months. This temporal criterium was established to maximize the likelihood of obtaining accurate information on the first phase of treatment. To minimize rater-dependent biases (i.e., therapist effects), each therapist was allowed to select only one patient. In addition, to ensure a random selection of patients, clinicians were asked to consult their appointment calendars to identify the last patient they had seen who met the study criteria. Despite acknowledging the role of the patient's perspective, in the present study, only the therapist's point of view was considered to focus on their contribution to session outcome in terms of depth of elaboration. Participation was voluntary and completely anonymous to guarantee privacy. All the clinicians provided informed consent. The research protocol was approved by the Ethical Committee of the Department of Dynamic and Clinical Psychology, and Health Studies, Faculty of Medicine and Psychology, Sapienza University of Rome, Rome, Italy, Protocol number 000073, date of approval 12/05/2022.

#### 2.1.1 Therapists

This sample comprised 84 White therapists, 38 males and 46 females. Their main age was 46 years approximately (*SD* = 10.36; range = 30–65). The average length of their clinical experience was 14.34 years (*SD* = 9.62, range = 2–43). The weekly hours devoted to clinical practice were approximately 30.26 (*SD* = 13.10, range = 6–55). The main clinical-theoretical approach was psychodynamic (*N* = 73), whereas a minority portion was cognitive-behavioral (*N* = 10).

#### 2.1.2 Patients

This sample comprised 84 White patients, 59 females, and 25 males. Their mean age was 35 years approximately (*SD* = 11.97, range = 20–65). Forty-four patients had only a DSM-5 personality diagnosis: one patient had a Cluster A disorder (diagnosed with a paranoid personality disorder); 18 had a Cluster B personality disorder (five diagnosed with a borderline personality disorder, four with a histrionic personality disorder, and nine with a narcissistic personality disorder); 14 had a Cluster C personality disorder (two diagnosed with an avoidant personality disorder, seven with a dependent personality disorder, and five with an obsessive-compulsive personality disorder); 16 patients presented two or more personality disorder diagnoses (eight had a comorbidity within the Cluster B and four a comorbidity within the Cluster C; four had a comorbidity between different clusters); 35 patients had personality disorder with or without other specification. The average length of treatment was about 8.38 months (*SD* = 6.44; range = 2–12).

### 2.2 Measures

#### 2.2.1 Patient's experience of attunement and responsiveness scale—therapist version

PEAR (Snyder and Silberschatz, 2017) is an instrument developed to assess clinician attunement and responsiveness in psychotherapy sessions from therapist (PAER-T) and patient (PEAR-P) perspectives. The PEAR-T consisted of 22 items that revealed a two-factor structure: (a) *therapist helpfulness*, which describes the therapist's perception that the patient found his/her interventions and attitude helpful and (b) *safe accepted*, which describes the therapists' impression that the patient felt safe with, and accepted by the therapist. The clinician assesses each item on a 5-point Likert scale ranging from 0 (*not at all) to 3 (very much)*. Only the therapist version (PEAR-T) was employed in the present research. As in the study of Snyder and Silberschatz (2017), the PEAR-T scale (ω = 0.78) and its subscales therapist helpfulness (ω = 80) and safe accepted (ω = 75) demonstrated good reliability.

#### 2.2.2 Depth scale of the session evaluation questionnaire

The SEQ-D (Stiles and Snow, 1984b; Rocco et al., 2017) is an instrument designed to assess the depth of elaboration in psychotherapy sessions from patient's and therapist's perspectives. It consists of five items that are scored on a 7 point-Likert scale. The items represent bipolar adjectives describing specific features of the session (i.e., powerful/weak, valuable/worthless, deep/shallow, full/empty, and special/ordinary). This scale showed good psychometric proprieties (Rocco et al., 2017), which are confirmed in this research in terms of reliability (Cronbach's α is 0.75).

#### 2.2.3 Therapist response questionnaire

The TRQ (Betan et al., 2005; Tanzilli et al., 2016) is a 79-item clinician-report questionnaire developed to evaluate the therapist's emotional responses (e.g., countertransference) in terms of thoughts, feelings, and behaviors toward the patient. The items are written in a jargon-free language to be understandable to clinicians of different theoretical orientations. The clinicians assess each item on a 5-point Likert scale, ranging from 1 (*not true*) to 5 (*true*). The factor structure of the Italian version showed nine countertransference patterns: (a) helpless/inadequate, indicates feelings of inadequacy, incompetence, and inefficacy; (b) overwhelmed/disorganized, describes confusion, anxiety, and intense feelings of being overwhelmed by the patient's emotions and needs; (c) positive/satisfying, describes an experience of close connection, trust, and collaboration with the patient; (d) hostile/angry, describes feelings of anger, hostility, and irritation toward the patient; (e) criticized/devalued, describes a sense of being criticized, dismissed, or devalued by the patient; (f) parental/protective, captures a wish to protect and nurture the patient in a parental way; (g) special/overinvolved, indicates that the patient is very special, so much so that the clinician may show some difficulties in maintaining the boundaries of the therapeutic setting; (h) sexualized, describes the presence of sexual attraction toward the patient; and (i) disengaged, describes feelings of annoyance, boredom, or withdrawal in sessions. The Italian validation of the TRQ demonstrated excellent psychometric properties. In this study, the nine TRQ dimensions showed good/excellent internal consistency (Streiner, 2003), obtaining the following Cronbach's alpha: criticized/devalued (α = 0.78), helpless/inadequate (α = 0.90), positive/satisfying (α = 0.82), parental/protective (α = 0.72), overwhelmed/disorganized (α = 0.77), special/overinvolved (α = 0.70), sexualized (α = 0.81), disengaged (α = 0.81), and hostile/angry (α = 0.84).

### 2.3 Statistical analysis

Data analyses were performed using JAMOVI version 2.4.11, with the application of jAMM statistical package (including the GLM mediation model module) (Gallucci, 2021). First, a multivariate analysis of covariance (MANCOVA) was used to investigate differences between two groups of psychotherapy sessions with poorer vs. greater degree of depth of elaboration (assessed with the SEQ-D) in therapist responsiveness (assessed with the PEAR-T) and specific patterns of clinician emotional reactions (assessed using the TRQ), after controlling for the impact of treatment duration. Notably, depth levels of psychotherapy sessions were distinguished by considering the median value of the SEQ depth scale in the total sample (*N* = 84). Thus, in MANCOVA, the groups of sessions with high or low depth of elaboration were used as the independent variable, all the therapist and relational dimensions as dependent variables, and psychotherapy length as a covariate.

Then, following the approach of Baron and Kenny (1986), a mediation analysis was carried out to test the potential mediator role of positive countertransference pattern in the relationship between therapist responsiveness (i.e., the average of the scores of the two dimensions of the PEAR-T) and depth of elaboration (considered as a continuous variable) in the psychotherapy sessions. In this model, the effect of therapy duration was also controlled for. This mediation analysis was conducted using the bootstrap percentile method. It was employed to construct the 95% confidence intervals to assess the statistical significance of these effects (Hayes and Rockwood, 2017). These bootstrap 95% confidence intervals (with 5,000 samples) were calculated to evaluate if they included zero.

### 2.4 Procedures

Clinicians were asked to choose one patient in their care according to inclusion and exclusion criteria (see “Participants sampling” for the description). After a psychotherapy session with the selected patient, they completed an online survey (hosted on SurveyMonkey), including: the PEAR-T, TRQ, and SEQ-D.

## 3 Results

### 3.1 Therapist responsiveness and countertransference patterns affecting depth of elaboration in psychotherapy sessions

The first aim of the study was to investigate the differences between psychotherapy sessions characterized by different degrees of depth of elaboration (evaluated with the SEQ-D) on therapist responsiveness (evaluated with the PEAR-T) and various countertransference patterns (evaluated using the TRQ).

One-way MANCOVA was used to determine whether psychotherapy sessions with lower vs. higher levels of depth of elaboration (distinguished based on the median value of SEQ depth scale of 4.80) were significantly affected by specific dimensions of clinician responsiveness and distinct patterns of therapist emotional responses, after removing the impact of treatment duration (Table 1). The results showed significant main effects for session groups, Wilks's λ = 0.65, *F*_(11, 71)_ = 3.51, *p* < 0.001, η^2^ = 0.35, while no significant effect was found for treatment duration, Wilks's λ = 0.89, *F*_(11, 71)_ =0.82, *p* < 0.621, η^2^ = 0.113.

**Table 1:** Differences between groups of psychotherapy sessions with different levels of depth of elaboration on therapist responsiveness and countertransference patterns after controlling for treatment duration (*N* = 84).

	**Deeper sessions (*****N*** = **46)**		**Shallower sessions (*****N*** = **38)**		***F*_(1, 81)_**	**η^2^**
	***M***	***SD***	***M***	***SD***		
**PEAR -T** ^a^					
Therapist helpfulness	2.90	0.41	2.63	0.34	10.62^**^	0.116
Safe accepted	2.62	0.28	2.53	0.29	1.70	0.021
**TRQ** ^b^					
Criticized/devaluated	1.36	0.29	1.54	0.62	3.41	0.040
Helpless/inadequate	1.61	0.58	2.18	0.93	10.58^**^	0.116
Positive	3.21	0.64	2.66	0.51	19.76^***^	0.196
Parental/protective	2.79	0.73	2.81	0.80	0.02	0.001
Overwhelmed/disorganized	1.65	0.45	1.60	0.53	0.23	0.003
Special/overinvolved	1.47	0.41	1.40	0.52	0.57	0.007
Sexualized	1.37	0.60	1.24	0.45	1.01	0.012
Disengaged	1.50	0.48	1.94	0.76	9.57^**^	0.106
Hostile/angry	1.60	0.49	1.91	0.69	4.70^*^	0.055

^a^PEAR-T, Patient's Experience of Attunement and Responsiveness Scale—Therapist Version (Snyder and Silberschatz, 2017). ^b^TRQ, Therapist Response Questionnaire (Tanzilli et al., 2016).

^*^p <0.05. ^**^p <0.01. ^***^p <0.001.

Notably, the deeper sessions differed significantly from the shallower sessions with respect to the higher levels of therapist helpfulness and positive countertransference. Moreover, these sessions, characterized by great depth of elaboration, showed significantly lower levels of helpless/inadequate, disengaged, and hostile/angry therapist responses than the shallower ones.

### 3.2 Therapist responsiveness, positive countertransference, and depth of elaboration in psychotherapy sessions: a mediation model

**Figure 1:**
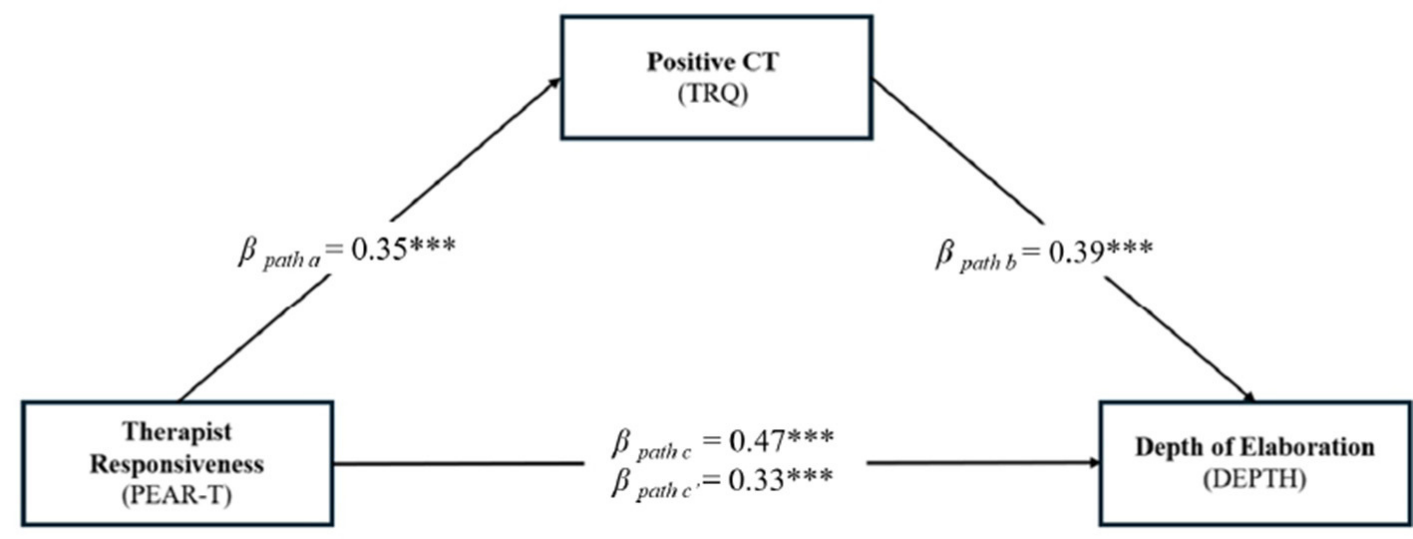
A mediation model examining the direct and indirect effect of therapist responsiveness on depth of elaboration in psychotherapy sessions through positive countertransference (*N* = 84). PEAR-T, Patient's Experience of Attunement and Responsiveness Scale—Therapist Version (Snyder and Silberschatz, 2017); TRQ, Therapist Response Questionnaire (Tanzilli et al., 2016); DEPTH, Depth Scale of Session Evaluation Questionnaire (SEQ) (Stiles and Snow, 1984b; Rocco et al., 2017). The figure includes completely standardized path coefficients (*betas*) obtained using a series of multiple regressions to construct the mediation model. ***p ≤ 0.001.

The study's second aim was to examine the mediation role of positive countertransference in the relationships between therapist responsiveness and depth of elaboration in psychotherapy sessions, controlling for the effect of treatment duration (Figure 1).

The results are also reported in Table 2. Overall, the mediation analysis showed that therapist responsiveness had a significant indirect effect on the depth of elaboration through the pathway of positive countertransference, ß = 0.1378 (95% C.I. 0.09746, 0.062026), *z* = 2.691, *p* = 0.007. Notably, the clinicians' positive response partially mediated the relationship between therapist responsiveness and depth of sessions, accounting for 29% of the total variance.

**Table 2:** Indirect and total effects in the mediation analysis including countertransference pattern as mediator in the relationship between therapist responsiveness and depth of elaboration in psychotherapy sessions (*N* = 84).

				**95% C.I**.				
**Type**	**Effect**	**Estimate**	**SE**	**Lower**	**Upper**	β	**Z**	***p***
Indirect	PEAR-T ⇒ positive CT ⇒ DEPTH	0.35886	0.13337	0.09746	0.62026	0.1378	2.691	0.007
Component	PEAR-T ⇒ positive CT	0.78085	0.22463	0.34058	1.22111	0.3544	3.476	<0.001
	Positive CT ⇒ DEPTH	0.45958	0.10814	0.24764	0.67153	0.3888	4.250	<0.001
Direct	PEAR-T ⇒ DEPTH	0.86690	0.23810	0.40022	1.33357	0.3328	3.641	<0.001
Total	PEAR-T ⇒ DEPTH	1.22576	0.24687	0.74190	1.70962	0.4706	4.965	<0.001

PEAR-T, Patient's Experience of Attunement and Responsiveness Scale—Therapist Version (Snyder and Silberschatz, 2017); DEPTH, Depth Scale of Session Evaluation Questionnaire (SEQ) (Stiles and Snow, 1984b; Rocco et al., 2017); CT, Countertransference Pattern of Therapist Response Questionnaire (Tanzilli et al., 2016).

## 4 Discussion

The primary aim of the present study was to examine differences in therapist responsiveness and countertransference patterns between sessions with distinct degrees of depth of elaboration in the treatment of individuals with personality disorders, regardless of the duration of treatment. The results partially confirmed our hypothesis (Table 1), showing that clinicians tended to exhibit higher levels of attunement and responsiveness in “good” sessions, characterized by greater depth of elaboration (Hatcher, 2015; Elliott et al., 2018). It is important to note that significant differences were observed in the dimension of *therapist helpfulness* but not in that of *safe accepted*. This finding suggests the crucial role of therapists' interpersonal skills, particularly their ability to be close to the patient and make them feel helped and supported during sessions (Timulak, 2010; Heinonen and Nissen-Lie, 2020; cf., Liotti et al., 2023). Additionally, therapists' perceptions of providing timely interventions and noticing patients' relief, success, and progress appear to correlate with valuable exchanges (Hatcher, 2015; Kramer and Stiles, 2015; Elliott et al., 2018; Wu and Levitt, 2020). Notably, this relational skill can be especially relevant for therapists treating individuals with personality disorders who show maladaptive interpersonal patterns that can hinder their engagement in the therapeutic work (Kramer, 2021; Culina et al., 2023). These results are in line with previous qualitative studies, which emphasize the importance of the therapist's sensitivity to the moment-to-moment state of the patient (Levitt and Piazza-Bonin, 2011; Kleiven et al., 2022; Ladmanová et al., 2022). Levitt and Piazza-Bonin (2011) also highlighted the relevance of therapist empathy, honesty, and validation, Kleiven et al. (2022) found that helpful therapist actions, such as “*actively helping the clients to notice and stay with difficult experience,”* were related to a greater likelihood of engaging in profound topics. Castonguay et al. (2010b) pointed out the detrimental effect of clinicians' failure to attune to the patient's needs and communication, particularly concerning issues that can trigger strong reactions such as interpersonal conflicts, further underscoring these therapist capacities. Furthermore, the present study suggested that patient's feeling safe was not directly associated, from the therapist's perspective, with the exploration of meaningful subjects in psychotherapy sessions. Snyder and Silberschatz (2017) indicated that the dimension of safe accepted (and not therapist helpfulness) was strongly related to the patient's evaluation of the treatment outcome; therefore, the result from our empirical investigation may confirm that this dimension is more closely associated with a broader inclusive representation of the treatment, rather than the evaluation of the individual session.

Looking at the specific and nuanced findings provided in Table 1, some relevant considerations need to be addressed on countertransference patterns. Consistent with our hypothesis, the results revealed significant differences in therapists' emotional responses between sessions with higher and lower levels of depth of elaboration, regardless of treatment duration. Specifically, only the positive countertransference pattern was significantly more prevalent in “good” sessions, whereas helpless/inadequate, disengaged, and hostile/angry responses recurred more in “bad” sessions characterized by lower levels of depth. Contrary to the study by Rocco et al. (2021), which did not identify any relationship between self-report countertransference evaluations and depth of elaboration, our study highlighted such a connection, suggesting considering the role of therapist constellations of thoughts, behaviors, and feelings (that is, their emotional responses) in influencing session quality (Rosenberger and Hayes, 2002; Ulberg et al., 2014; Abargil and Tishby, 2022).

The presence of positive therapist responses in deeper sessions supports the conceptualization of countertransference as a potentially valuable tool for gaining meaningful insights into the patient's needs and not as an obstacle to the session process (Gabbard, 1998; Tishby and Wiseman, 2014; Tanzilli and Lingiardi, 2022; Pellens et al., 2023). Positive countertransference encompasses affiliation and emotional closeness (Tanzilli et al., 2016). Interestingly, research showed that it is linked with the therapeutic alliance, further supporting its connection with an atmosphere of collaboration and trust in therapeutic work (Ulberg et al., 2014; cf., Tanzilli and Gualco, 2020).

Regarding the clinician's negative emotional responses, the study revealed that sessions with lower levels of depth of elaboration were strongly characterized by a greater degree of helpless/inadequate, disengaged, and hostile/angry countertransference patterns. These findings underscore the negative association between these intense and difficult-to-manage therapist reactions and the exploration of meaningful issues (Tishby and Wiseman, 2014; Abargil and Tishby, 2022). They are aligned with previous studies that have emphasized the potentially harmful effect of negative countertransference reactions, particularly when they are not effectively managed (Ulberg et al., 2014; Hayes et al., 2018; Tanzilli et al., 2018). Feelings of hostility, boredom, or helplessness can divert the therapist's focus away from the patient's emotional state (Rosenberger and Hayes, 2002; Gross and Elliott, 2017; Rocco et al., 2021) thereby interfering with the fundamental condition necessary for an emphatic and attuned elaboration process (Hennissen et al., 2023; Pellens et al., 2023). These specific therapist emotional reactions have been observed in the treatment of patients with personality pathologies, who often manifest maladaptive interpersonal patterns, such as devaluating and dismissing attitudes (Colli et al., 2014; Tanzilli et al., 2017). Therapists working with these patients may experience significant difficulties in reaching a high depth of elaboration in the therapeutic process. Finally, it should be acknowledged that these reactions of detachment or irritation might indicate underlying ruptures in the therapeutic alliance (Safran et al., 1990; Safran and Kraus, 2014). Therapists' awareness of their own feelings toward patients, as well as empathic resolution strategies, are essential tools for preserving the quality of the therapeutic relationship and facilitating in-depth exploration of the patient's inner dynamics (Eubanks-Carter et al., 2015; Tishby and Wiseman, 2022).

The second aim of the present study was to examine the mediating role of positive countertransference in the relationship between therapist responsiveness and session depth. Consistent with the hypotheses, this study provided preliminary confirmation of this mediation model (Table 2). Therapist responsiveness and attunement were found to be systematically associated with greater depth of elaboration (cf., Harrington et al., 2021), but positive countertransference played a key role by shedding light on some mechanisms through which therapists promote a profound elaboration of content in psychotherapy sessions. Presumably, therapists' subjective experience of a positive relationship with patients, characterized by intimacy, affective closeness, cooperation, and trust, enables them to be attuned and responsive to patients, promoting the working through of meaningful topics (cf., Hatcher, 2015; Snyder and Silberschatz, 2017). This study seems consistent with previous research that has shown how the clinician's positive, hopeful, and genuine emotional reactions were able to influence the quality of the therapeutic process (see Tishby and Wiseman, 2022), while negative reactions toward the patient can produce severely detrimental effects (Ulberg et al., 2014).

The present study has some limitations. First, the sample size might limit the generalizability of the findings, suggesting the need to replicate the present study on larger samples. Second, due to the research design's cross-sectional nature, it is not possible to establish causal relationships between the dimensions investigated in the study. Different study designs (i.e., longitudinal) should be employed to overcome this limitation. Third, the data collection method from a single informant (i.e., the clinician) might be vulnerable to biases. Further research should consider other perspectives, such as that of the patient or external observer. Fourth, the present study only considered patients with a personality disorder diagnosis, limiting the generalizability of these findings and suggesting the need to replicate this study within different clinical populations. Moreover, most of the clinicians were psychodynamic, and their theoretical orientation may have influenced the study results. Future research should investigate the effect of the therapist's clinical background on the dimensions of therapeutic relationship and process investigated in this study to shed light on possible associations underlying the quality of psychotherapy sessions and, potentially, treatment outcomes. Finally, treatment outcomes were not considered in the present study, indicating a need for further research to address this gap.

## 5 Conclusions

The present study emphasizes the importance of acknowledging therapists' contribution to the individual session outcome considering the effect of their interpersonal capacities that develop during the clinical encounter. Particularly, treating patients with personality disorder diagnoses (Hatcher, 2015; Johns et al., 2019; Heinonen and Nissen-Lie, 2020), who may potentially impact therapeutic work with challenging behaviors, seems to require especially timely and appropriate responsiveness from the clinician in order to achieve greater session quality (Kramer et al., 2014; Signer et al., 2020; Kramer, 2021). It further suggests the need to identify moments of misattunement during clinical work—giving particular attention to the therapist's perception of being helpful—and to address negative countertransference reactions (e.g., detachment, inadequacy) that can hinder a profound elaboration of the content of the psychotherapy session. On the contrary, the value of the therapist's capacity to be attuned and responsive to the patient's needs, along with the presence of trustful and collaborative feelings to facilitate the exploration of meaningful content, is advocated (Kramer and Stiles, 2015; Snyder and Silberschatz, 2017).

## Data Availability

Data supporting the findings of this study are available from the corresponding author on request.
